# The correlation between the high-intensity zone on a T2-weighted MRI and positive outcomes of discography: a meta-analysis

**DOI:** 10.1186/s13018-017-0523-1

**Published:** 2017-02-08

**Authors:** Chunyang Fang, Wenbin Zhang, Liqiu Chen, Hongjie Li

**Affiliations:** Department of Orthopedics, The First People’s Hospital of Wenling, No. 190, Taiping Nan Road, Wenling, Zhejiang Province 317500 People’s Republic of China

**Keywords:** Low back pain, MRI, High-intensity zone, Discography, Meta-analysis

## Abstract

**Background:**

This meta-analysis aimed to assess the correlation between the high-intensity zone (HIZ) of a lumbar MRI and discography.

**Methods:**

We conducted an electronic search of the PubMed, MEDLINE, Embase, and ScienceDirect databases from their respective inceptions to October 2016 using the following search terms: “low back pain,” “discogenic low back pain,” “HIZ or high-intensity zone,” and “discography”. Relevant journals and conference proceedings were manually searched. Two reviewers independently assessed the quality of the studies, extracted data from the included studies, and analyzed the data.

**Results:**

Eleven studies were included. The results of the meta-analysis indicated that outstanding relativity and statistically significant correlations were observed between the HIZ and abnormal disc morphology (OR = 47.79; 95% CI: 17.07 to 133.77; *P* < 0.00001), HIZ and pain reproduction (OR = 8.65, 95% CI: 6.01 to 15.23, *P* < 0.00001), and HIZ and abnormal morphology pain reproduction (OR = 11.74, 95% CI: 1.99 to 69.36, *P* = 0.007).

**Conclusions:**

The presence of an HIZ on a lumbar MRI T2-weighted image indicates abnormal disc morphology. There is a strong relationship between the HIZ and pain reproduction. The HIZ can be an effective index for prediction of discogenic low back pain.

## Background

Low back pain (LBP) is a common and devastating condition that causes disability or other severe complications [1]. In recent years, the incidence of LBP has gradually increased, and provocative discography is considered the gold standard for diagnosing LBP [2]. However, provocative discography is invasive and associated with complications, including neurological injury, infection, or contrast medium reaction [3].

In 1992, Aprill and Bogduk [4] first described a high-intensity zone (HIZ) on magnetic resonance imaging (MRI) located in the posterior annulus fibrosus that was clearly separated from the nucleus pulposus. The appearance of an HIZ may indicate the rupture of intervertebral disc fibrous rings, which would cause nucleus pulposus herniation along the fissure. Leaked nucleus pulposus leads to an inflammatory reaction, which can result in the accumulation of granulation tissue with neovascularization as a reparative response. This phenomenon was observed on an MRI T2 as an HIZ and on discography as morphological abnormalities of the intervertebral disc. Several studies have confirmed that HIZ showed a notable histologic feature of the formation of vascularized granulation tissue and may be a specific indicator for an inflammatory reaction [5, 6]. Aprill and Bogduk demonstrated that in morphologically abnormal discs, a significant correlation exists between an HIZ-positive disc and exact or similar pain reproduction on provocative discography.

The subsequent literature has reported consistent results [7, 8]; however, several studies have shown a limited role of the HIZ in diagnosing LBP [9–11]. Consequently, the correlation between HIZ-positive discs and exact or similar pain reproduction on provocative discography remains controversial. Moreover, a few limitations, such as small sample size, inaccurate evaluations, and deficiencies in study design, could be observed in previous studies. Therefore, we conducted a large-scale meta-analysis to assess the correlation between an HIZ on lumbar MRI and provocative discography.

## Methods

### Search strategy

Based on the Cochrane Collaboration guidelines, we searched electronic databases including Cochrane Library, MEDLINE (1966–October 2016), PubMed (1966–October 2016), Embase (1980–October 2016), and ScienceDirect (1985–October 2016). In addition, the reference lists of all included studies were manually searched to identify trials that may have been missed in the initial search.

**Fig. 1:**
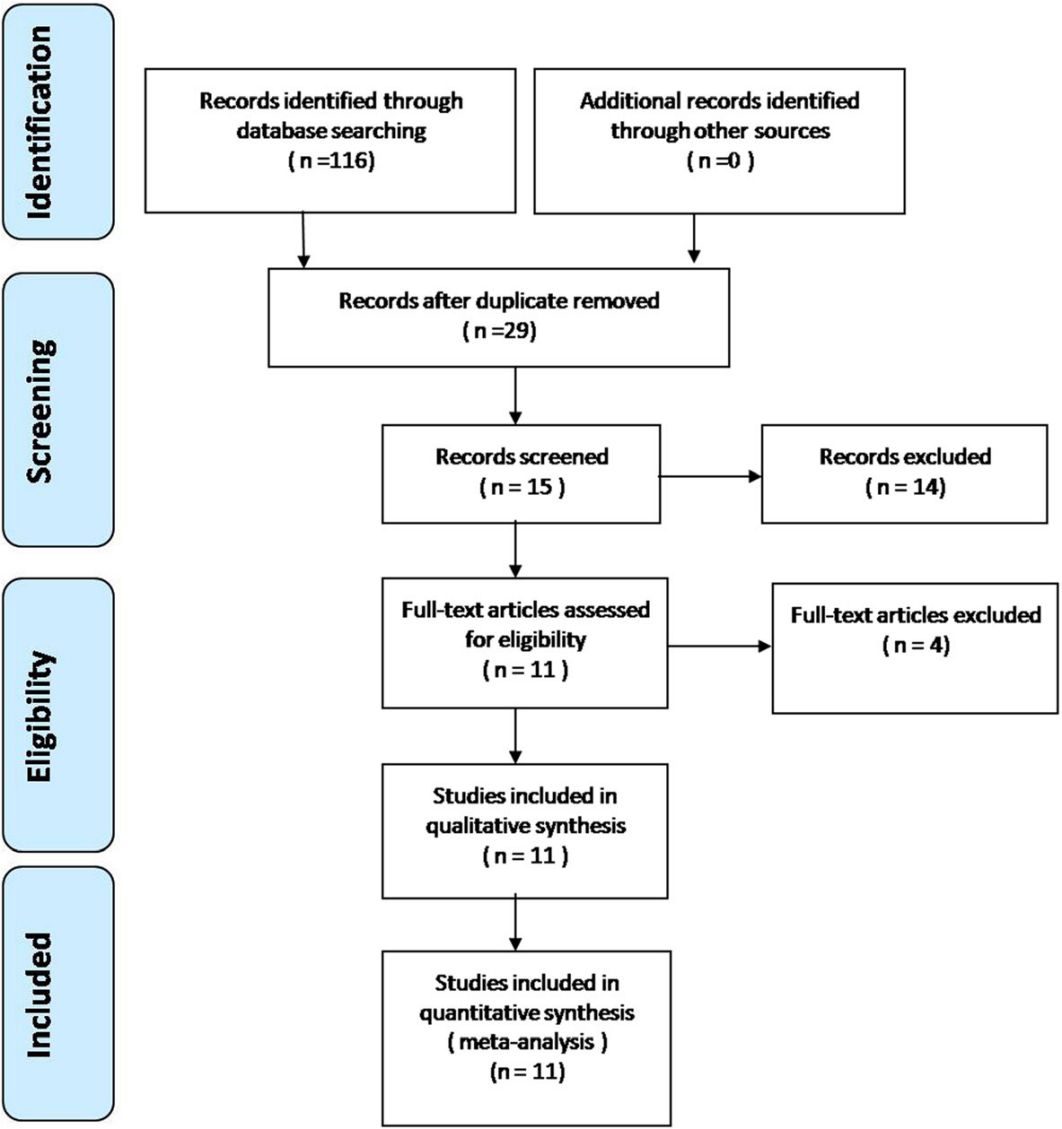
Flowchart of the study selection process

The search process was conducted as shown in Fig. 1. We used the keywords “low back pain,” “discogenic low back pain,” “HIZ or high-intensity zone,” and “discography” in combination with the Boolean operators AND or OR. This study is a meta-analysis, which does not require either an ethics committee or institutional review board to approve the study.

### Inclusion criteria

This review was conducted in accordance with guidelines described in the Cochrane handbook for systematic review and meta-analysis of interventions [12] and met the criteria of the Meta-analysis of Observational Studies in Epidemiology (MOOSE) [13].

Two review authors screened the titles and abstracts of all studies identified by the search strategy. Then, we retrieved the studies for full-text review and re-evaluated the articles according to the following inclusion criteria: (1) LBP assessed by MRI and discography and (2) exploration of HIZs on MRI and morphologically abnormal discs or similar pain reproduction on provocative discography. There were no restrictions on the patients’ gender and age or time of publication. Disagreements were resolved by consensus.

### Quality assessment

The methodological quality of the included studies was assessed by two independent reviewers according to the Strengthening the Reporting of Observational Studies in Epidemiology Statement (STROBE) [14]. The methodological quality of the studies was classified into three levels: A: more than 80% conformation to the STROBE standard; B: between 50 and 80% conformation to the STROBE standard; and C: less than 50% conformation to the STROBE standard. Any disagreements were resolved by either consensus or consultation with a third reviewer.

### Data extraction

Statistical analysis was performed using Review Manager 5.2 software (The Cochrane Collaboration, Oxford, United Kingdom), and a *P* value <0.05 was considered statistically significant. For each eligible study, we calculated the odds ratios (OR) for dichotomous variables with 95% confidence intervals (CI). If outcomes were measured in the same way between studies, we calculated the mean differences (MD) and 95% CI for continuous variables. Heterogeneity of the mean differences across the studies was assessed using the chi-squared test and *I* ^2^ statistic. If the results were significant (*P* < 0.1 or *I* ^2^ > 50%), a random effects model was used to estimate the overall effect sizes, and a sensitivity analysis was performed to investigate the potential sources of heterogeneity. Otherwise, a fixed effects model was adopted. Moreover, publication bias among the studies was assessed by funnel plots.

## Results

### Literature search

The study selection process is shown in Fig. 1. We identified a total of 116 articles with our search strategy. After removing duplicates, scanning titles and abstracts and reading the full text, we identified 11 studies that were eligible based on our inclusion criteria [4, 5, 9, 15–22].

### Study characteristics and quality assessment

All included trials involved lumbar discs and were published in English. The detailed characteristics of the studies are displayed in Table 1. Nine of included studies were considered A level, and two of included studies were considered B level [15, 18].

**Table 1:** Cohort characteristics

Author	Study design	Magnetic field intensity	Was discography method described?	Did the observer of MRI and discography know each other?	Is the statistical method correct?	Methodologic quality
Aprill 1992	CS	0.6 T	Yes	No	Yes	A
Schellhas 1996	RA	1.5 T	Yes	No	Yes	A
Ricketson 1996	CCT	Not stated	Yes	No	Yes	B
Saifuddin 1998	RA	0.5–1.5 T	Yes	No	Yes	A
Ito 1998	CCT	1.5 T	Yes	No	Yes	A
Smith 1998	RS	1.5 T	Yes	No	Yes	A
Lam 2000	RS	1.5 T	Yes	No	Yes	A
Carrage 2000	RA	Not stated	Yes	No	Yes	B
Lim 2005	CCT	1.5 T	Yes	No	Yes	A
Peng 2006	CCT	1.5 T	Yes	No	Yes	A
Chen 2011	CCT	1.5 T	Yes	No	Yes	A

*CS* cohort study, *RA* retrospective analysis, *CCT* case-control study

### Outcomes of the meta-analysis

#### The relationship between HIZ and morphology in provocative discography

**Fig. 2:**
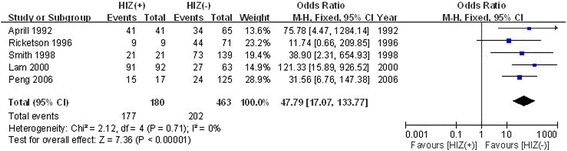
Forest plot diagram showing the relationship between the HIZ and morphology based on provocative discography

The Dallas discogram scale [23] was used to evaluate the morphology based on provocative discography. Five included studies reported the relationship between HIZ and morphology based on provocative discography. No significant heterogeneity was found; therefore, the fixed effects model was applied (*χ* ^2^ = 2.12, *df* = 4, *I* ^2^ = 0, *P* = 0.71). In the pooled analyses, there was a significant difference in morphologically abnormal discs (OR = 47.79; 95% CI: 17.07 to 133.77; *P* < 0.00001) between the HIZ-positive disc group and the HIZ-negative disc group (Fig. 2).

#### The relationship between the HIZ and pain reproduction based on provocative discography

**Fig. 3:**
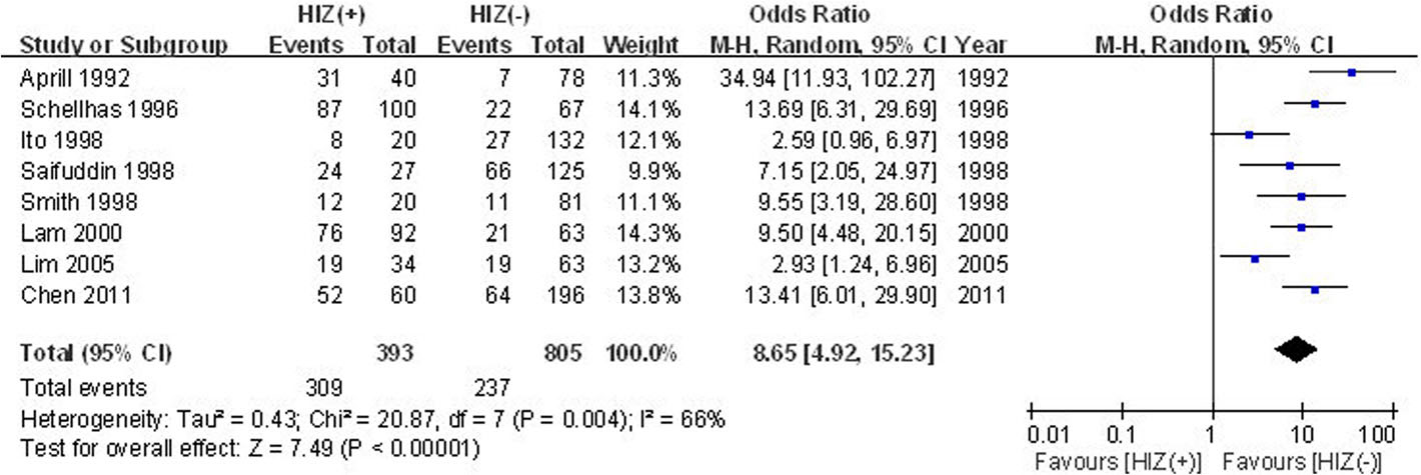
Forest plot diagram showing the relationship between the HIZ and pain reproduction based on provocative discography

The relationship between the HIZ and pain reproduction based on provocative discography was shown in eight studies. Significant heterogeneity was found; thus, the random effects model was applied (*χ* ^2^ = 20.87, *df* = 7, *I* ^2^ = 66%, *P* = 0.004). There were significant differences between the two groups (OR = 8.65, 95% CI: 6.01 to 15.23, *P* < 0.00001; Fig. 3).

#### The relationship between the HIZ and pain reproduction of discs with abnormal morphology

**Fig. 4:**
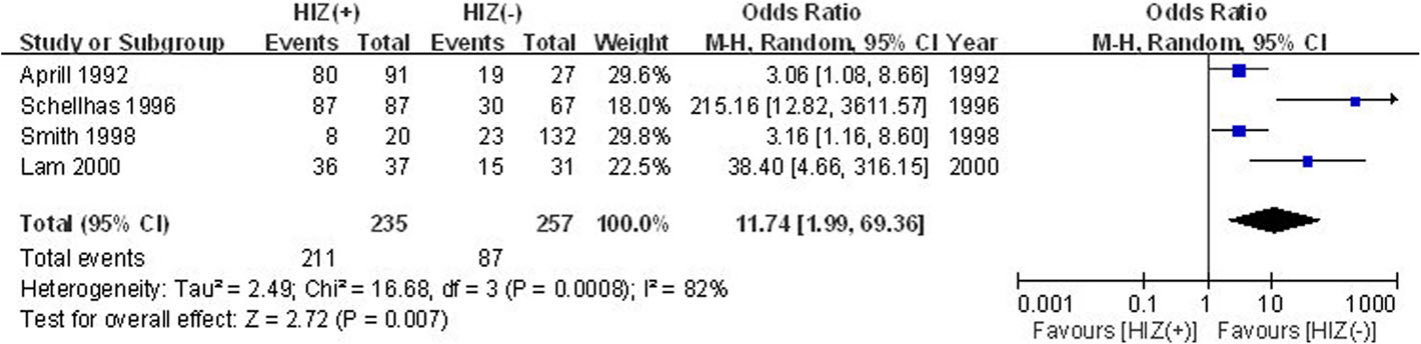
Forest plot diagram showing the relationship between the HIZ and pain reproduction of discs with abnormal morphology

The relationship between the HIZ and pain reproduction of discs with abnormal morphology was described in four studies. Significant heterogeneity was found; thus, the random effects model was applied (*χ* ^2^ = 16.68, *df* = 3, *I* ^2^ = 82%, *P* = 0.0008). Pain reproduction of discs with abnormal morphology in the HIZ-positive disc group was significantly higher than that in the HIZ-negative disc group (OR = 11.74, 95% CI: 1.99 to 69.36, *P* = 0.007; Fig. 4).

## Discussion

To our knowledge, this is the first meta-analysis to compare the HIZ with other metrics of LBP, which can help us obtain a more precise assessment of this phenomenon. The most important finding of the present study was that the HIZ on a lumbar MRI T2-weighted image was associated with abnormal disc morphology in the discography. In addition, there was a significant relationship between the HIZ and pain reproduction. The findings of the present study have important implications as they indicate that HIZ is a highly effective parameter in determining the intensity of discogenic LBP.

In 1992, Aprill and Bogduk first proposed that the HIZ can be a valuable indicator of a ruptured lumbar disc leading to LBP [4]. The appearance of an HIZ may indicate the rupture of intervertebral disc fibrous rings, which would cause nucleus pulposus herniation along the fissure. Leaked nucleus pulposus results in an inflammatory reaction, which can lead to accumulation of granulation tissue with neovascularization as a reparative response. This phenomenon was observed on an MRI T2 as an HIZ and on discography as morphological abnormalities of the intervertebral disc.

Trauma has been suggested as one of the causes of intervertebral disc disruption (IDD) [5]. Annular tear could possibly originate from trauma because of the structurally weak posterior part of annulus fibrosus [24]. However, Park et al. concluded that the presence of HIZs on MR images showed a very weak correlation to trauma [25].

Several studies have investigated the clear association between the HIZ and provocative discography. The published literature has shown that the incidence of HIZ in patients with LBP is 25–50% [16, 20]. Schellhas et al. [20] reported that 87 of the 100 investigated HIZ discs were concordantly painful at the time of discography. Ito et al. [16] assessed the MRIs of 39 patients with LBP, including 101 discs, and found that 60% of the HIZ-positive discs (12/20) were concordantly painful at the time of discography, whereas 11% of the HIZ-negative discs (11/81) were concordantly painful at the time of discography. Lam et al. [9] concluded that there was a significant correlation between the HIZ and either exact or similar pain reproduction in morphologically abnormal discs. Similar findings were demonstrated in other studies [4, 26].

In contrast, a previous study by Ricketson et al. [18] suggested that the HIZ was not related to the annular disruption in discography. Lei et al. [27] reported that the sensitivity and specificity of the HIZ were 27 and 87%, respectively. Saifuddin et al. [19] confirmed that the HIZ is a marker of a painful posterior annular tear, but the usefulness of this parameter is limited by its low sensitivity (26.7%). However, all of the abovementioned studies had relatively small sample sizes and were limited by insufficient statistical power. Thus, the exact association was still unclear.

In the present study, 11 studies of high methodological quality were included in this meta-analysis, and all of them met the MOOSE and STROBE requirements. In 2016, Jha et al. [3] performed a review of the HIZ for discogenic LBP. However, they did not extract data for further quantitative analysis. Thus, we conducted the present meta-analysis including all published studies to precisely estimate the relationship between the HIZ and provocative discography.

There were several potential limitations that should be noted. (1) The sample sizes of the included studies were relatively small; (2) the methodologies of the included studies have their own limitations; and (3) a subgroup analysis was not performed because we were unaware of any sources of heterogeneity due to limited number of included studies.

## Conclusions

In conclusion, this meta-analysis showed that the presence of an HIZ on a lumbar MRI T2-weighted image indicates abnormal disc morphology. There is a strong relationship between the HIZ and pain reproduction. Finally, the HIZ can be an effective parameter to determine the contribution of the disc in LBP.

## Abbreviations

CI: Confidence intervals
HIZ: High-intensity zone
IDD: Intervertebral disc disruption
LBP: Low back pain
MD: Mean differences
MOOSE: Meta-analysis of Observational Studies in Epidemiology
MRI: Magnetic resonance imaging
OR: Odds ratios
STROBE: Strengthening the Reporting of Observational Studies in Epidemiology Statement
